# Multinuclear MRI in Drug Discovery

**DOI:** 10.3390/molecules27196493

**Published:** 2022-10-01

**Authors:** Dorota Bartusik-Aebisher, Zuzanna Bober, Jolanta Zalejska-Fiolka, Aleksandra Kawczyk-Krupka, David Aebisher

**Affiliations:** 1 Department of Biochemistry and General Chemistry, Medical College of Rzeszów University, 35-310 Rzeszów, Poland; 2 Department of Photomedicine and Physical Chemistry, Medical College of Rzeszów University, 35-310 Rzeszów, Poland; 3 Department of Biochemistry, Faculty of Medical Sciences in Zabrze, Medical University of Silesia, 40-055 Katowice, Poland; 4 Center for Laser Diagnostics and Therapy, Department of Internal Medicine, Angiology and Physical Medicine, Medical University of Silesia in Katowice, 41-902 Bytom, Poland

**Keywords:** tracking, MRI, drug cell culture tissue

## Abstract

The continuous development of magnetic resonance imaging broadens the range of applications to newer areas. Using MRI, we can not only visualize, but also track pharmaceutical substances and labeled cells in both in vivo and in vitro tests. ^1^H is widely used in the MRI method, which is determined by its high content in the human body. The potential of the MRI method makes it an excellent tool for imaging the morphology of the examined objects, and also enables registration of changes at the level of metabolism. There are several reports in the scientific publications on the use of clinical MRI for in vitro tracking. The use of multinuclear MRI has great potential for scientific research and clinical studies. Tuning MRI scanners to the Larmor frequency of a given nucleus, allows imaging without tissue background. Heavy nuclei are components of both drugs and contrast agents and molecular complexes. The implementation of hyperpolarization techniques allows for better MRI sensitivity. The aim of this review is to present the use of multinuclear MRI for investigations in drug delivery.

## 1. Introduction

Magnetic resonance imaging (MRI) has been one of the most popular and accurate diagnostic methods over the past few decades [1]. MRI enables the examination of the entire human body in a non-invasive way, without the use of harmful ionizing radiation. About 24 elements are essential for life: H, C, N, O, F, Na, Mg, Si, P, S, Cl, K, Ca, V, Mn, Fe, Co, Ni, Cu, Zn, Se, Mo, Sn and I [2]. MR is utilized in spin (*I* = ½) nuclei such as ^1^H, ^13^C, ^15^N, ^31^P, and 3/2 nuclei such as ^23^Na, and is performed in liquid solutions or solid materials. MRI is used to assess many anatomical tissues and is used in clinical diagnostics to image structures of the body such as the central nervous system [3], heart [4], muscles [5] and bone tissue [6]. The anatomical structure and biochemical composition of the examined tissue result in different signal intensities in MRI images [7,8]. The use of MRI in the tracking of nuclei in targeted tissue is beneficial for developing new theranostics procedures and optimizing drug delivery [9]. The role of drug delivery to tissue is to increase the concentration of a specific drug in a tissue region of interest with minimal nontargeted distribution. Thus, drug delivery provides a high level of therapeutic efficacy in targeted tissue [10]. The usefulness of multinuclear MRI is proven in tracking labeled compounds in vitro or in vivo. Also, MRI is a non-invasive technique that allows monitoring during time course studies. Current MRI systems have parallel imaging capability (due to more than one channel). The second heteronuclear transmitter and a range of different radiofrequency coils allows not only standard ^1^H MRI but also ^13^C, ^19^F, ^23^Na or ^31^P MRI and spectroscopy [11]. MRI can answer important biological questions in preclinical and clinical research. MRI has the potential to contribute substantially to drug delivery research in the pharmaceutical industry. The configuration of MRI apparatus allow the use of this machine in in vivo research in neurology, cardiology, oncology, infectious diseases, endocrinology, immunology, stem cell imaging, virtual pathology and others. 

The MRI guided drug delivery technique not only helps reduce nontarget distribution of the drug but also increases drug concentration in the targeted area. MRI guided drug delivery can be used to quantified and also visualize drug in real-time settings. In clinical research, MRI guided drug delivery techniques belongs with high intensity focused ultrasound (HIFU), which is an emerging non-invasive method of MRI guided drug delivery [12]. HIFU uses ultrasonic radiation to heat the tumor, which causes necrosis, hence, temperature-sensitive carrier molecules, such as liposomes, micelles, or polymers release the drug while passing through it. To the MRI guided drug delivery technique belongs MRI imaging used to visualize the concentration of super-paramagnetic iron oxide (SPIO)-based nanoparticles as a nanosystem candidate for the diagnosis and treatment of cancer. 

Multinuclear MRI—also called nonproton MRI—has recently garnered gathering interest with the increased availability of ultra-high-field MRI systems. Assuming the availability of a broadband RF amplifier, performing multinuclear MR experiments essentially requires additional hardware, such as an RF resonator and a T/R switch for each nucleus [13]. 

The enrichment of macromolecules in stable isotopes allows for the dispersion of ^1^H, ^13^C, and ^15^N chemical shifts into multiple spectral dimensions in a manner that preserves the chemical and/or spatial relationship between atoms within a molecule of interest.

Multinuclear spectroscopy table of nuclides currently used in human MRI and MRS studies is shown in Table 1 [14].

This review is organized as follows: the first section provides an overview of MRI emphasizing the practical applications related to use of MRI in drug delivery. The second part discusses the physical phenomenon of magnetic resonance, the third part is related to compound tracking in vitro and the fourth to in vivo tracking. The last section describes the future opportunities of multinuclear MRI used in drug tracking and the conclusion.

## 2. The Physical Phenomenon of Magnetic Resonance

### 2.1. MRI Sequences

**Figure 1:**
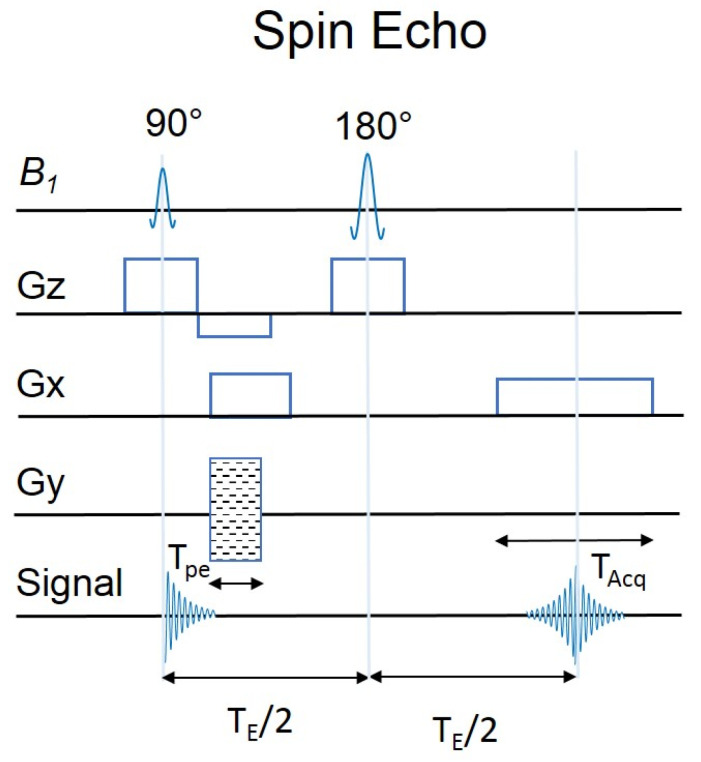
Scheme of SE pulse sequences. T_pe_ is the duration of the gradient. T_Acq_ is the duration of signal acquisition. B_1_ is an Radio Frequency energy field applied perpendicular to the longitudinalaxis (B_0_).

There are several types of sequences in MRI. The basic spin echo (SE) sequence is characterized by the excitation of spins by sending a series of radio frequency (RF) pulses. As shown in Figure 1, we have 90° and 180° RF pulses (Figure 1). They are both needed to produce SE. The 90° RF pulse is designed to excite and bridle the signal from the region of interest along with a layer selection gradient (Gz). Gz is applied with the appropriate amplitude to be able to excite a layer of a certain thickness. The Gx gradient is a frequency-encoding gradient, while the Gy gradient encodes phase. The gradients are run in a periodic order to produce echoes and obtain lines in the raw data space (K-space). Subsequently, a second RF pulse is sent to reverse the direction of the precession. SE is often used in clinical research, particularly to study the skeletal, muscular and head systems. The fast spin echo (FSE) sequence uses the same principle. However, in this sequence, several 180° pulses are sent simultaneously for repetition (TR), and the signal is measured several times. First a 90° pulse is sent, then a 180° pulse is sent, after the expiration (time to echo) of the TE signal measurement, then a 180° pulse is sent and the signal is measured repeatedly, after the TR expires another 90° pulse is sent. Another sequence used is STIR (inversion recovery), which allows the ability to “turn off” the signal from some tissues. It involves first sending a 180° pulse to invert the magnetization. Then a 90° activation pulse is sent after the inversion time (TI), which is the time between the magnetization reversal pulse and the excitation pulse. The value of the TI time depends on the magnetic field and the type of tissue. For example, to measure fat, TI = 150 ms at a magnetic field of 1.5 Tesla is used. At the time of measurement, the fat will not send a signal and, as a result the image will be dark [15].

**Figure 2:**
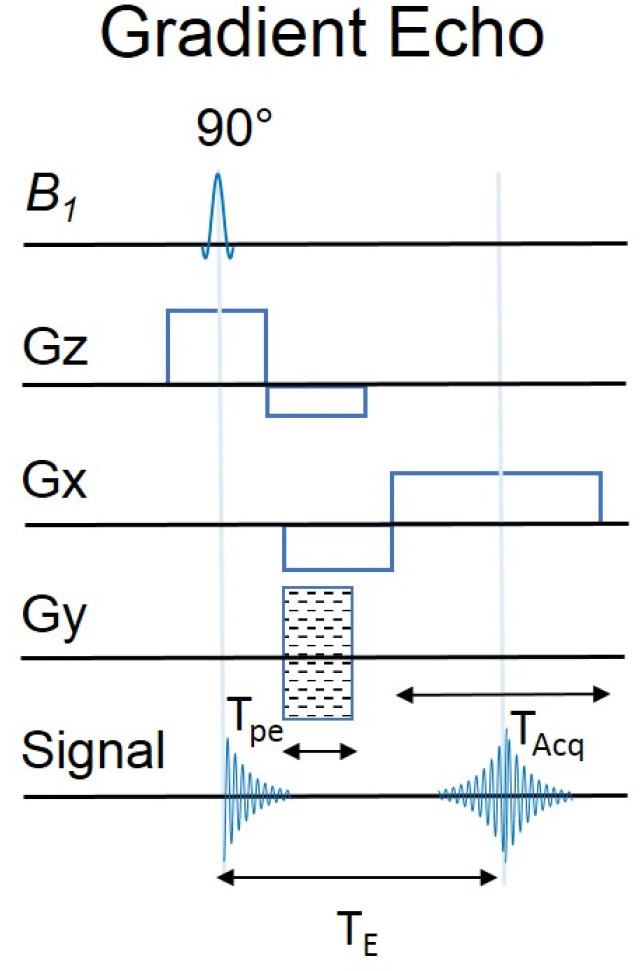
Scheme of GE pulse sequences. T_pe_ is the duration of the gradient. T_Acq_ is the duration of signal acquisition. B1 is an Radio Frequency energy field applied perpendicular to the longitudinalaxis (B0).

The gradient echo (GE) sequence is similar in structure to the SE sequence, except that the pulse orientation does not change by 180° (Figure 2). During the measurement, the direction of the gradient changes, after the stimulating pulse the protons change direction, thanks to the switched gradient poles. In the case of GE, we are dealing with a radiofrequency (RF) pulse that deflects the spin through an angle less than or different from 90°. The GRE pulse sequence is very similar to SE, differing only in the changes in the Gx gradient and the missing 180° RF pulse. These seemingly small differences are responsible for producing gradient echoes. In GRE, the first negative Gx gradient is a de-phasing gradient, de-phasing the magnetization vector in the transverse plane. Subsequently, the application of positive gradients is to out-phase the spins, so as to usher in a perfectly focused gradient echo during TE. A Gy phase encoding gradient, is used to create echo lines in the raw data space. Equally, the layer selection gradient Gz, is designed to excite the layer of interest during the initial RF pulse [16].

When both types of sequences are used, a current is induced in the receiving coil, which is then converted into a digital signal stored in a matrix valid in k-space. The image matrix (MTX) contains phase and spatial frequency information and is then processed using the Fourier transform (FT) to produce an image. In SE, an additional 180° pulse helps eliminate magnetic field inhomogeneities. The 180° pulse, omitted from GE sequences, does not compensate for these inequalities. Transverse magnetization relaxes not with a constant T_2_, but with a constant T_2_*. T_2_* also depends on the inhomogeneity of the external magnetic field, and this causes a chemical shift to occur. GE sequences are faster and more resistant to artifacts. In the clinic, they are used for abdominal and thoracic examinations, as well as for angiography. Some sequences use both transverse and longitudinal magnetization, these are (Fast Imaging Employing Steady-State Acquisition) FIESTA and true Fast Imaging with Steady-State Precession) FISP used for cardiac examination [17,18].

### 2.2. T_1_ and T_2_ MRI Relaxation Times

The signal intensity (SI) for a single voxel from the MTX consists of three factors, such as: spin density, spin lattice relaxation time (T_1_) and spin–spin relaxation time (T_2_) for the material tested. SI is also significantly influenced by the choice of TE and TR parameters. As soon as the impulse is removed, the protons return to the ground state, giving back the accumulated energy. Due to the heterogeneity of the magnetic field, as well as interactions between protons, they are decoded. The signal measured from de-phased protons can be weak. Therefore, an additional 180° impulse is used, which changes the direction of the protons in such a way that the faster ones are at the bottom (they have a longer path to travel) and the slower ones are at the top (they have a shorter path). At the time of measurement, the spins are in the same phase. We can distinguish two types of magnetization: longitudinal and transverse. T_1_ relaxation is also called spin-lattice relaxation or longitudinal relaxation, it is the time required for the water protons to return to their ground state after applying the 90° RF pulse. The longitudinal component of the total magnetic nuclear moment vector decreases exponentially until it reaches equilibrium. This is the time needed for the magnetization component Mz to recover about 63% of its original value. On the other hand, T_2_ relaxation (transverse magnetization) is defined as the time it takes for the spins to lose their coherence with each other. It is the relaxation of the transverse component (perpendicular to the external magnetic field) of the nuclear magnetization vector. T_1_ is used to study the strength and topology of intermolecular interactions, such as drug–drug, drug–protein, drug–DNA, drug–micelle (or vesicle) and biomolecule–biomolecule interactions. The use of T_1_ time determination methods can be helpful for drug design and evaluation [19].

The phenomenon of MR has a number of applications in clinical diagnostics in advanced imaging techniques such as: magnetic resonance spectroscopy (MRS) [20], diffusion (DWI), diffusion tensor imaging (DTI) [21], magnetic susceptibility (SWI) [22], perfusion (PWI) [23] and functional magnetic resonance imaging (fMRI) [24], is also used for experimental studies such as controlled release dosage forms, hydration and diffusion, labeling of cells with nanoparticles [25] and real-time metabolic imaging [26].

MRI is a commonly used method for evaluating inflammation and is characterized by high sensitivity. The high resolution of the image and the specificity of the device make it possible to visualize inflammation in the human body. It detects at an early-stage rheumatoid arthritis, ankylosing spondylitis, infectious spondylitis, encephalitis, meningitis, myocarditis and other inflammatory diseases. MRI has the potential to be used to assess the inflammatory response. Studies show visualization of elastin after myocardial infarction using an elastin-binding contrast agent in a mouse model [27]. Other studies show quantification of the inflammatory response after allergen provocation in allergic rhinitis [28].

### 2.3. Variable MRS Methods

MRS is a diagnostic tool for assessing chemical composition [28,29,30,31]. MRS is used to determine the chemical properties of an area, focusing on cell metabolites. The method is based on the effect of the chemical shift of the atom, the nuclei of various cells pass at different frequencies [32,33]. MRI allows you to locate anatomical structures, while MRS is used to assess the chemical composition of the tissue. The most frequently performed experiment is single voxel spectroscopy (SVS), where the signal is received from a selected location. Measurements are performed using the PRED (pointed-resolved spectroscopy) or STEAM (stimuled echo aquisition mode) sequence. On the basis of the recorded signal from a given voxel, the Fourier transform is calculated, and then spectra are generated, in which individual peaks react to individual metabolites [34]. Based on the amplitude of the signal, a plot of the chemical frequency shift of the signal in parts per million (ppm) is generated. The area under the peak corresponds to the concentration of the metabolite. This makes it possible to quantify the signal using internal standards. In clinical trials, MRS allows for the identification and quantification of metabolites, including: N-acetyl-l-aspartic acid (NAA), creatine (Cr), choline (Cho) and lactate (Lac).

Using MRI, we have a tool that is helpful in understanding the processes involved in drug metabolism. This could have a significant impact on the development of a new generation of drugs. MRI can identify tissue macromolecules such as nucleic acids, lipids, collagen and proteoglycans using parameters such as chemical shift, relaxation rates and magnetic spin coupling. With MRS, the analysis time is short and multiple metabolites can be analyzed during a single measurement. In contrast, other methods are slightly more time-consuming and require different chromatographic techniques depending on the metabolites being studied. MRS is more costly in terms of equipment and its maintenance, but has a low or zero cost in terms of sample/patient preparation for testing. There are many methods used for compound identification, such as high-pressure liquid chromatography (HPLC), nuclear magnetic resonance (NMR) spectroscopy, microscopy, thin-layer chromatography (TLC), gas chromatography–mass spectrometry (GC–MS), ultra-pure liquid chromatography (UPLC), Fourier transform infrared spectroscopy (FTIR), liquid chromatography quadrupole time-of-flight mass spectrometry (LCMS-TOF) and high-performance thin-layer chromatography (HPTLC). Studies using MRS can be performed in vivo, without prior preparation. For example, the sample preparation process in MS is more complex and tissue extraction is also required

### 2.4. Multinuclear MRI

Multinuclear MRI allows imaging without a tissue background, which is proving to be extremely important for pharmaceutical research. Among other things, they enable monitoring of drug delivery, assessment of metabolism, tracking of labeled cells and the ability to visualize novel drug conjugates.

Magnetically abundant nuclei such as ^1^H, ^19^F, ^31^P and others are excellent for multinuclear MRI experiments [35]. The ^1^H nucleus has a spin equal to ½ and due to its high content in the human body and having a high value gyromagnetic coefficient, it is the most frequently imaged element in MR, allowing images to be obtained with high resolution T_1_- or T_2_-weighting. One of the most popular nuclei used for tracking is ^19^F, which was used for a drug delivery study in breast cancer cells in vitro [36,37,38].

Numerous NMR studies present the detection of multiple free induction decays (FIDs) during a single survey, which allows for more spectral information and also allows for improved sensitivity. This has allowed for an increase in signal-to-noise ratio (SNR). Clinical scanners can be used successfully for multinuclear MRI. A typical scanner used for clinical applications with a 1.5 Tesla or 3 Tesla field has 32 to 64 receiver channels [39]. The higher the number of receiver channels, the faster the scanning. For multinuclear scans, multiple channels are used to improve SNR. Multinuclear studies, are also conducted at a low field of 0.5 Tesla for imaging proton, deuterium, fluorine, sodium, oxygen, boron, carbon, chlorine, silicon and phosphorus. Signal attenuation algorithms have been developed, allowing MRI images to be filtered. This enabled a series of pull-out studies of intravenously injected Perftoran^®^ blood substitute in small animal in vivo studies [32]. Multinuclear MRI was also used to detect early changes in the Schwannoma microenvironment (VS) after atrial radiosurgery. A study was conducted on five patients using 1.5 Tesla ^1^H and ^23^Na MRI. Sodium bioscales highlighted early changes in VS and an increase in tumor TSC as measured by ^23^Na MRI, detectable at 2 weeks after radiotherapy [40]. Other studies have used ^1^H/^23^Na MRI 7 Tesla to detect intracellular and extracellular components of TSC in breast cancer in vitro and in vivo studies on patients. The potential of using MRI to determine intracellular sodium concentration (CIC), extracellular volume fraction (ECV) was confirmed [41].

### 2.5. Hyperpolarized MRI/MRS

The use of hyperpolarized (HP) carbon-13 (^13^C) for MRI purposes is a new method of molecular imaging. It is especially valuable for metabolic imaging, allowing the study of a variety of biochemical processes. It is characterized by low abundance, hence difficulties in its imaging. However, the use of dynamic nuclear polarization technique allows the ^13^C signal to be increased. It is non-radioactive and safely enables real-time imaging and pathway-specific study of dynamic metabolic and physiological processes.

Quantitative and accurate monitoring of tumor response to treatment makes hyperpolarized ^13^C MRI/S a powerful tool for in vivo metabolic study and provides the opportunity for the implementation of hyperpolarized contrast agent. Moreover, the studies of the properties and functions in tumor tissue of the compounds of carbon that are organic, are fundamental to tumor biochemistry.

In nature, C is abundant in all forms of life and all dead organic materials. About 1.11% of naturally occurring C is ^13^C which is magnetically active and can be applied to probe molecular structures that correspond to physiological changes in tumor tissues. More than 98.89% of naturally occurring C is ^12^C (six protons and six neutrons) with no MR signal. Although the MR signal of ^13^C in vivo corresponds to metabolic changes, SI of naturally occurring ^13^C is too low to be relevant for quantitative and longitudinal studies. However, the visualization of ^13^C nuclei concentration may result in images and spectra with high SNR due to the hyperpolarization process. Hyperpolarization does not change any chemical or physical properties of the substances, however, it allows acquisition of ^13^C images and spectra in a relevant time frame. Hence, hyperpolarized ^13^C MRI/S can directly inform about the biochemical tissue composition and chemical structure by generating frequency and spatial distribution of hyperpolarized atoms. In general, most suitable ^13^C compounds for MR are small molecules (molecular weight ~120 gmol^−1^) with a possibility to obtain information about molecular behavior in vivo due to rapid uptake in tissue.

We review the applications of ^13^C hyperpolarized techniques such as para-hydrogen-induced-hyperpolarization (PHIP) and dynamic-nuclear-polarization (DNP) to monitor tumor targeting giving an overview of rapid ^13^C MRI/S sequences used which is followed by the hardware enhancement such as coil design. The examples of the main component of pulse sequences used for hyperpolarized ^13^C MRI/S are shown in Table 2.

Also, the use of ^31^P hyperpolarized nuclei allows for increased signal and real-time imaging of biological perfusion, metabolite transport and metabolic reactions in in vivo studies. This overcomes the limitations of conventional low-sensitivity MRI.

Dynamic Dissolution Nuclear Polarization (d-DNP) is a versatile method to overcome the limitations of nuclear MRS. It can increase SI by up to four to five orders of magnitude. Among other uses, it is used in metabolism, cellular studies, and real-time monitoring of chemical or biological processes. Studies show assessment of tissue metabolism in redox-related conditions such as cancer, inflammation and neurological disorders. It also enables treatment monitoring as a theranostic tool [47]. ^13^C are used in hyperpolarized studies, but [1-^13^C]pyruvate is most commonly used in clinical trials. Studies have shown that it enables detection of intracellular production of [1-^13^C]lactate and ^13^C-bicarbonate. It can image the Warburg effect in malignancies and detect features of ischemia or viability in the myocardium [48]. Hyperpolarized ^13^C MRI is a potential tool in detecting liver pathology, predicting disease progression, and monitoring applied therapies [49]. A promising technique for clinical use is spin exchange optical pumping (SEOP). In this technique, circularly polarized light is used to selectively optically pump (usually) rubidium (Rb) electrons, generating a highly spin-polarized Rb gas. A wide range of nuclei ^2^H, ^3^He, ^13^C, ^15^N, ^31^P, and ^129^Xe can be polarized [50]. The ^129^Xe hyperpolarization method can be used for lung imaging amongst others [51]. Another method is brute-force polarization. It involves achieving high polarization of the thermal equilibrium by applying a strong magnetic field and low temperature [52].

Multi-MRS enables the determination of many metabolic activities and reflects the actual metabolism in vivo. The high potential of the method can be complemented by the latest research in the field of molecular biology, biochemistry and metabolism studies. Hyperpolarized ^13^C MRS enables real-time measurement of enzymatic activity in living organisms. The method has so far most commonly been used to study cancer and heart disease. The use of hyperpolarized ^13^C and ^31^P MRS has contributed significantly to the understanding of glucose and phospholipid metabolism [53]. In addition, multi-MRS is being used for cardiac studies, the use of ^1^H, ^13^C and ^31^P provides a wealth of metabolic information for accurate diagnosis and treatment [54]. Hyperpolarized ^13^C MRI has the potential to assess tissue redox status, oxidative stress, and inflammation and cellular metabolism [55]. It also examines the branching points of metabolic pathways to quantify the fate of metabolites in acute lung injury and inflammation [56]. More recently, the method has also been used to study neurological disorders [57,58]. ^31^P MRS has potential for use in studies in hepatopancreatobiliary cancer [59].

**Figure 3:**
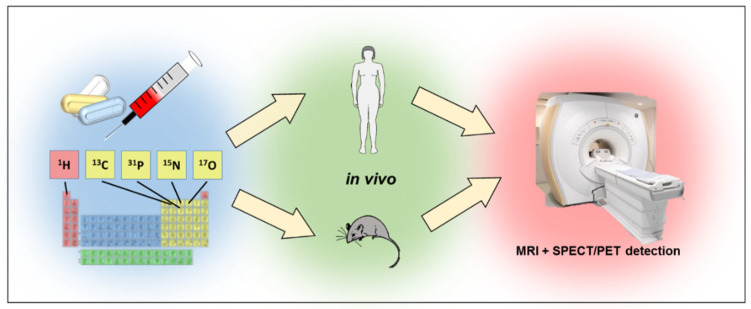
Using MRI to trace pharmaceuticals with different elements.

The hydrogen nucleus has a spin of ½, and because of its high content in the human body and high gyromagnetic coefficient value, it is the most commonly imaged element in MR, allowing for high-resolution T_1_- or T_2_-weighted images. Although MRI and MRS can use such nuclides as ^13^C, ^15^N, ^19^F, ^23^Na and ^31^P, ^1^H is the primary one in clinical diagnostics. In MRI, the signal sources are water and fat protons, while in MRS the signals come from metabolites. Herein we present the most important applications of various elements such as proton ^1^H, carbon ^13^C, nitrogen ^15^N, oxygen ^17^O and phosphorus ^31^P in in vitro and in vivo research (Figure 3).

## 3. Compound Tracking In Vitro

### 3.1. ^1^H MRI

^1^H is widely used in the MRI method, which is determined by its high content in the human body. There are several reports in scientific publications on the use of clinical **^1^H** MRI for in vitro tracking. Giesel et al. evaluated gadofluorine M with other gadolinium chelates for T_1_-weighted positive enhancement for in vitro cell tracking using a MR apparatus with 1.5 T magnetic field. The use of ultrafine iron particles to track cells in MRI is well known. However, the experimental models presented here have limitations related to intrinsic iron signals from erythrocytes, which interfere with imaging of labeled cells [60]. In other studies, Tang et al. successfully carried out an in vitro MRI study on porcine bone marrow stem cells labeled with SPIO-poly-L-lysine (PLL) and green fluorescent protein (EGFP) [61]. On the other hand, Feng et al. conducted research on a phantom with cartilage damage using mesenchymal stem cells (MSCs) labeled with SPIO. MRI was used to track SPIO labeled MSCs [62]. Addicott et al. in their research, presented a new contrast agent Molday ION Rhodamine-B ™ for labeling cells with MRI 1.5T in an in vitro study [63]. On the other hand, Freichels et al. in their research, using ^1^H NMR, they assessed the polylactide-co-glycolide (PLGA) polymer with a covalently linked fluorescent dye, maintaining the macromolecular properties of the polymer [64]. Also, Henning et al. performed MRI of mesenchymal stem cells labeled with ferumoxide in cartilage defects in in vitro and in vivo studies [65]. In the next presented studies, Lu et al. labeled rat bone marrow mesenchymal stem cells with polylysine coated SPIO (PLL-SPIO). In vitro MRI studies with PLL-SPIO has the potential to be a MRI tracking agent for tracking transplanted stem cells [66]. In other in vitro studies, rabbit mesenchymal stem cells were labeled with SPIO. On the basis of the performed studies, it was found that MSCs labeled with SPIO retain the ability to differentiate in vitro [67]. By contrast, Shuai et al. reported the tracking of Gd-DTPA labeled human umbilical cord mesenchymal stem cells (hUCMSC) by NMR [68]. Tang et al. developed a method for labeling bone marrow mesenchymal stem cells of a diabetic miniature Tibetan pig. The cells were labeled with various concentrations of SPIO and enhanced LV-eGFP green fluorescent protein, then followed by MRI in in vitro studies for 6 weeks [69]. On the other hand, Li et al. presented studies on the use of a non-yenne gadolinium contrast agent for the labeling of stromal cells in neonatal rats in in vitro studies. In addition, studies on the paramagnetic contrast agent showed detectability by MRI for 28 days [70]. On the other hand, the group of Geng et al. presented an in vitro study on the tracking of gadolinium-labeled diethylenetriamine pentaacetate (Gd-DTPA) mesenchymal stem cells in an in vitro cerebral ischemia model. Higher signal intensity in labeled cells was observed, while no apparent negative effect on cell viability or proliferation was observed [71]. Liu et al. used photostable fluorescent nanoparticles to track mesenchymal stem cells (MSCs) used in regenerative medicine. A system based on the PCL-DPP-PCL polymer complex was used, and it has been shown that it can be used for long-term monitoring of cells in the differentiation of adipogenic and chondrogenic MSCs [72]. On the other hand, Zhang et al. in their research presented the use of graphene oxide (GO) as a contrast agent for the determination of human mesenchymal stem cells (hMSCs). GO-DOTA-Gd complexes were prepared, which in vitro tests at 11.7 T showed better T_1_ relaxation than the popular contrast agent Magnevist [73]. Lu et al. presented new contrast medium microparticles—PLGA/iron oxide (MP PLGA / IO MP) used for MRI tracking [74]. Attention should be paid to regenerative therapies based on cell transplants, which are aimed at treating various types of chronic disorders and diseases of the heart muscle [75], post-stroke lesions [76], degenerative joint diseases [77] to restore normal physiological functions. Monitoring the fate of the transplanted cells and checking the interaction with the environment in which they have been implanted turn out to be extremely important. In order to make such an assessment, they should be monitored in real time.

### 3.2. ^19^F MRI

The fluorine nucleus has a resonance frequency similar to the hydrogen frequency. In order to obtain a satisfactory signal in ^19^F MRI, it is necessary to obtain a very high density of ^19^F nuclei in the tested sample. The use of non-toxic and chemically inert fluorine nuclei is increasingly used in pharmaceuticals, in particular chemotherapeutic anesthesia, as well as in the substitution of blood and oxygen for respiration. Fluorinated compounds can be tested by ^19^F MRI and ^19^F MRS. Research on nanoparticle emulsions consisting of perfluoro-15-crown-5-ether (PFCE) or perfluoroacetyl bromide (PFOB) cores has been implemented. ^19^F MRI and ^19^F MRS studies confirm the feasibility of using a clinical scanner for molecular imaging. The ^19^F MRS spectrum can vary over a range of more than 100 ppm. ^19^F MRI is used to measure renal oxygen tension and blood volume [78]. Also in the research, probes based on DOTP chelate similar to Gd^3+^ chelates with 12 magnetically equivalent fluorine atoms (DOTP-tfe) and a lanthanide ion (III) were presented and tested. T_1_ and T_2_ relaxation times were measured at 4.7 Tesla, the effect of chelated lanthanide (III) ion on the reduction in relaxation times in in vitro tests was noted [79]. Zare et al. presented the tracing of pulpal stem cells of a SPION-labeled dextran-coated tooth pulp in in vitro studies. At doses lower than 25 mg/ml, no toxicity was found, and SPION labeling did not affect cell survival or differentiation, so they can be used in regenerative medicine [80]. Chirizzi et al. used fluorine probes and multispectral MRI to track the activity of immune cells. The tests were carried out using a three-dimensional sequence of fast spin echo with the use of ^19^F nanoparticles of two different fluorocarbons. The ^19^F MRI results showed high sensitivity and specificity of murine mononuclear cells both in vitro and in vivo [81]. On the other hand, Wang et al. used quantitative tracking of encapsulated CT and ^19^F MRI mesenchymal stem cells to assess transplant immunodetection. Measurements were made on phantoms and in vivo on rabbits. Measurements showed agreement between CT and MRI both in vitro and in vivo [82]. Also, Richard et al. conducted studies on the PFC labeling of human progenitor (hGRP) restricted (gGRP) cells (Q cells), developed optimized labeling protocols and showed that PFCs did not significantly alter the glial identity of Q cells [83]. Researchers developed novel Nafion-based nanocarriers for ^19^F MRI enabling imaging without tissue background [84]. The potential of iron-based metal–organic structures as a theranostic carrier for topical TB therapy is also presented. Studies show that the Fe-MIL-101-NH_2_ metal-organic structure (MOF) releases the drug inside cells. This is a novel approach in the strategy of delivering standard antitubercular agents combined with monitoring their distribution in lung tissue [85]. The researchers also presented research on an inhalable theranostic system for the topical treatment of tuberculosis containing a metal organic frame loaded with isoniazid Fe-MIL-101-NH2-From Raw MOF to Drug Delivery System [86]. Presented Fe_3_O_4_@SiO_2_ designed @Au nanoparticles for MRI-guided photothermal therapy of cancer cells. The fabricated particles exhibit very strong T_2_ contrast MRI properties and have the potential to be used in cancer photothermal therapy, which is simulated by irradiating two colon cancer cell lines [87].

### 3.3. ^31^P MRI

In addition, research on the ^31^P nucleus has been implemented in scientific research. Robinson et al. in their studies presented the use of ^31^P MRS for studies on various murine T40 fibrosarcoma, T115 breast cancer and T237 lung cancer transplants to evaluate the parameters of ^31^P-MRS. The aim of the study was to test the energy patterns and blood flow during growth, compared to the pattern of the faster growing RIF-1 fibrosarcoma [88].

Ouwerker et al. assessed errors in in vivo ^31^P MRS measurements in the selection of scanning parameters such as sequence repetition time [89]. In other studies, Martino et al. performed quantitative metabolic studies of phosphorylated drugs such as 5-fluorouracil, its prodrug capecitabine, 5-fluorocytosine, in mass solutions the 10-microM limit of quantification was determined for ^31^P NMR [90]. On the other hand, Landis et al. using ^31^P MRS, assessed hepatocytes transplanted into the liver in mice, which is an alternative to transplantation in the treatment of liver diseases [91]. Zhang et al. Group used the labeling of cells with superparamagnetic iron oxide nanoparticles to track cells using ^1^H MRS and ^31^P MRS for cell evaluation. The results show that it is possible to use ^31^P MRS to assess the viability of labeled therapeutic cells [92]. Cameron et al. assessed the content of intracellular magnesium using ^31^P MRI, determined the function of skeletal muscles and their aging in comparison with the content of magnesium in the serum [93].

### 3.4. ^15^N MRI

**Figure 4:**
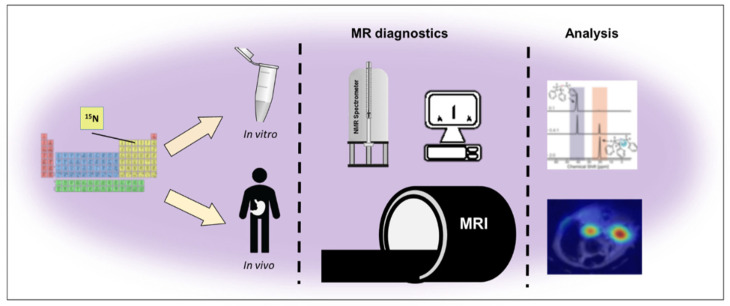
The scope of use of ^15^N MRI in research.

There are also reports in research studies on ^15^N both in vitro and in vivo (Figure 4). Gabellier et al. in their research, presented the use of dynamic nuclear polarization to determine the ^15^N MRI signal in four-fold enhanced choline, which is a precursor of cellular metabolism of phospholipids [94]. Chiavazza et al. proposed the use of ^15^N-permethylated amino acids as probes for innovative research into ^15^N MRI tracking [95]. On the other hand, Jagtap et al. presented research on perdeuterated molecules containing ^15^N, such as: tert-amines derivatives of aniline and quaternary pyridinium compounds with ^15^N, which reaches a polarization of ^15^N to 8%, due to its content in various drugs, it can be used in drug delivery systems. In addition, the use of hyperpolarization techniques may contribute to the improvement of the sensitivity of contrast agents used for MRI, extending their longitudinal T_1_ relaxation time [96].

## 4. Tracking In Vivo

### 4.1. ^1^H MRI

In recent years, we can see the development of in vivo research. The group of Cao et al. performed double banking of BMSC bone mesenchymal stem cells with USPIO and fluorescent protein (RFP). They were then incubated with the culture medium for 24 h and transplanted into the myocardium of rats and MRI performed in vivo [97]. Whereas Xu et al. presented a study on the tracking of USPIO and Siner-labeled poly-L-lysine (PLL) human umbilical cord mesenchymal stromal cells (HUMSCs) [98]. Agudelo et al. in in vivo studies, used Dex-DOTA-Gd3 (+) to track endothelial progenitor cells transplanted in rats with limb ischemia, and a signal was obtained that enabled EPC tracking [99]. Laughney et al. in in vivo studies in mice, investigated resistance to eribulin and a fluorescent analog. The use of intravenous imaging enabled the assessment of taxane resistance. The MDR1-mApple fusion protein fluorescently “differentiated” resistant cells, moreover, the MDR1 inhibitor encapsulated in a nanoparticle delivery system reversed the multidrug-resistant phenotype and enhanced the effects of eribulin in in vitro and in vivo studies in mice [100]. Constantinides et al. used ^1^H and ^19^F MRI to track labeled stem cells. Medium chain polyhydroxyalkanoates (MCL-PHA) were used, in vitro cytocompatibility studies were performed with perfluoro ether-nanoparticle labeled mouse CPCs and examined by confocal microscopy and ^19^F MRS and MRI. Based on the conducted research, it was concluded that the MCL-PHA / PCL mixtures in the future may be used in CPC delivery systems and improvement of regeneration in myocardial infarction [101]. Shahror et al. in their research described a new technique for labeling and tracing MSCs labeled with SPIO. The nanoparticles were loaded with fluorescein isothiocyanate (FITC) and then intranasally implanted into the brains of mice and imaged in real time by MRI [102]. In in vivo studies, Bardhan et al. followed the multimodal therapeutic nanocomplexes to evaluate the effect of HER2 antibody targeting the degradation of the nanocomplexes over 72 h. This allowed determination of their distribution and their subsequent fate [103]. Shan, on the other hand, described the use of labeled iron nanoparticles for labeling mesenchymal stem cells [104]. However, the studies by Al Faraj et al. used carbon nanotubes for non-invasive MRI tracking in a mouse breast cancer model for future use as a drug delivery vehicle. Carbon nanotubes (SWCNTs) can be traced by MRI due to their high sensitivity [105]. In contrast, Danhier et al. used the tracking of SP10-labeled cells by MRI and electron paramagnetic resonance (EPR) to visualize and evaluate murine breast cancer cells in the brain of mice. The use of these two techniques allowed for complementary cell tracking, both their detection by 11.7T MRI and the evaluation of cell numbers by EPR [106]. Another group, Hong et al., used magnetic nanowires to isolate and detect circulating tumor cells (CTCs) of 29 patients (100%) with early breast cancer without metastases [107]. Makela et al. quantified macrophages using MRI cell tracking labeled with iron oxide (USPIO) and perfluorocarbons (PFC). Based on the conducted research, it was found that fluoride labeling provides more information on the density of tumor-associated macrophages than iron labeling [108]. Due to their high sensitivity and accuracy, nanowires have great potential for tracking cells in medicine. On the other hand, Rammohan et al. developed a series of Gd (III) gold nanoconjugates for labeling breast cancer cells. Due to the diversified chelate structure and the length of the nanoparticle–chelate linker, it is possible to use this type of marker for MRI imaging. Moreover, they showed good tolerance in vivo, which additionally bodes well for future use for cell tracking in in vivo studies [109]. On the other hand, Murrell et al. studied the fate of iron-labeled cancer cells after radiotherapy using the MRI method to test the effect of cranial irradiation and the growth of breast cancer metastases to the brain in the MDA-MB-231-BR-HER2 human cell model [110]. The detection of macrophages indicates the presence of cancer cells or metastases. The use of this type of biomarker in the treatment of patients would enable the selection of the best treatment regimen and the monitoring of the progress of therapy. In subsequent studies by Brewer et al. magneto-endosymbionts (ME) were used to track cells in in vitro and in vivo studies, assessing their biomedical properties. In the case of ME, the relaxation value r2 was (250 s^−1^ mM^−1^), while for the conventional SPIO (178 s^−1^ mM^−1^). ME-labeled cells showed strong MR contrast [111]. In their research, the group of Martínez-Banderas et al. used magnetic nanowires with an iron core and an iron oxide coating as contrast agents for cell tracking by MRI. The concentration of 0.8 μg Fe/ml used made it possible to detect 25 cells/μL in in vitro tests, which enables the implementation of this type of imaging to track cell therapies [112]. The group of Ramm et al. in other studies, used the tracking of glioblastoma cancer stem cells (CSC) in an in vitro pilot study to evaluate biomarkers for clinical magnetic resonance spectroscopy. Ten CSC cell lines were examined by high resolution ^1^H-NMR at 14.4 and 18.8 Tesla. The spectra obtained were analyzed on the basis of the main component (PCA), which allowed distinguishing between samples with high and low clonogenicity [113]. On the other hand, Chen et al. labeled mesenchymal stem cells in vitro. The optimal PEI2k-SPIO threshold was determined, which was 7 μm/ml, after MRI examinations, no negative impact on the activity of stem cells was found, while obtaining clear MRI images [114]. On the other hand, the group of Xu et al. followed mesenchymal stem cells with poly (lactide-co-glycolide) microparticles loaded with iron oxide nanoparticles. They showed that the internalization of loaded IO-NP (10 nm) biodegradable poly (lactide-co-glycolide) microparticles (IO/PLGA-MP, 0.4-3 μm) in stem cells improved MR parameters compared to IO-NP alone, and thus does not threaten their viability, proliferation and migration, or the ability to accumulate in places of inflammation [115]. On the other hand, Herea et al. in their work presented an in vitro model of adipose tissue derived stem cells (ADSCs), using MNPS coated with palmitate (MNPsPA), which are to act as a carrier of an anti-cancer drug. After performing a series of magnetic field tests, the group concluded that the created model could be used as a drug carrier, which could also be used for MRI tracking [116]. Struys et al. presented the use of MRI for imaging dental pulp stem cells (hDPSC), which have the ability to self-renew. hDPSC were labeled by SPIO, assessing labeling efficacy at low concentrations. Extremely high labeling efficiency of the transplanted cells into the brain of mice was observed at 15 µg/ml, in combination with 0.75 µg/ml poly-L-lysine (PLL) [117]. Whereas the group of Ferrauto et al. used PARACEST paramagnetic contrast agents to label different cell populations. Yb- complexes were used to label murine macrophages (J774.A1) and Eu-HPDO3A to label melanoma cells (B16-F10). After analysis, it was found that paramagnetic agents have the same stability and pharmacokinetic properties in in vivo studies as the commonly used contrast agent Gd-HPDO3A (ProHance^®^) [118].

### 4.2. ^19^F MRI

The fluorine nucleus in in vivo studies is used in various types of neoplasms, both in diagnostic and spectroscopic studies involving ^19^F-labeled drugs. In 1977, perfluorocarbon compounds (PFCs) were used for the first time by ^19^F MRI. PFCs emulsify and can carry drugs. ^19^F MRI allows for imaging without a tissue background, which turns out to be extremely important in the case of research on pharmaceutical substances. They enable, inter alia, the assessment of metabolism, tracking of labeled cells, the possibility of visualizing new fluorinated drug conjugates and monitoring drug delivery. Research into perfluorocarbons (PFCs) is popular, which is similar in structure to, for example, alkanes, except that all hydrogen atoms are replaced with a fluorine nucleus. Fluorocarbons are chemical compounds used in ^19^F MRI. They are similar in structure to the alkanes present biologically. The most commonly used are PFCE (perfluoro-15-crown-5-ether), PERFECTA (1,3bis[[1,1,1,3,3,3hexafluoro2(trifluoromethyl)propan2yl]oxy]2,2bis[[1,1,1,3,3,3hexafluoro2(trifluoromethyl)propan2yl]oxymethyl]propane) and PFOB (perfluorooctyl bromide). PFCs are hydrophobic and lipophobic, and are most commonly used as emulsions of PFCs stabilized with surfactants or encapsulated in polymer nanoparticles. They are used for cell tracking, imaging inflammation, and monitoring drug delivery. In recent reports, we can read about the use of PFCs for in vivo oxygen transport [119]. Perfluorocarbons have also found use in cancer models as therapeutic agents targeting hypoxia [120]. Because microcirculation is impaired in solid tumors, oxygen delivery is reduced. Hypoxia restricts the production of reactive oxygen species thus blocking is the most important treatments. To overcome this limitation, PFC nanoemulsions are used to deliver oxygen to tissues and prevent the deficit [121]. Guo et al. in their work presented the use of phase-shifted ultrasound-activated perfluorocarbon nanodroplets for anti-cancer therapy [122]. Constantinides et al. implemented studies on cardiac progenitor stem cells (CPCs) and macrophages derived from mouse bone marrow, which were labeled with PFCE (perfluoro-corona-ether), followed by measurements with ^19^F MRI and MRS at 9.4 Tesla [123]. On the other hand, Jahromi et al. presented a study of ^19^F MRI imaging probes with a metallic chelate based on a PFC-soluble salicylipfdene-tris (aminomethyl) ethane core for detecting inflammatory macrophages in mice in vivo [124].

For ^19^F MRI studies, a satisfactory signal must be obtained on ^19^F MRI in the study sample with a very high density of ^19^F nuclei allowing imaging without tissue background. In this study, we presented the labeling of human HSC CD34 + cells with perfusorocarbon ^19^F MRI. Kislukhin et al. in their study presented β-diketones conjugated with linear perfluor-polyether (PFPE) as paramagnetic fluorinated aqueous emulsions for tracking cellular therapies and inflammatory cells in vivo using ^19^F MRI. By improving the detection sensitivity of ^19^ F MRI by three to five times compared to previous tracers used at 11.7 T, sensitivity is expected to increase by about eight times for a 3 T magnetic field [125]. Chapelin et al. in their work presented perfluorocarbon probes for ^19^F MRI used in the study of resistance cell therapies used in anti-cancer therapies [126]. The group of Makela et al., using ^19^F MRI presented in vivo studies of cell tracking and macrophage distribution in mouse breast cancer tumors [127]. In another study, a group of hypertensive TGR(m-Ren2)27 rats were administered small doses of curcumin in hyaluronic acid-based nanocapsules, which induced a hypotensive effect in hypertensive rats. The nanocapsules were based on hyaluronic acid (HyC12-Cur), a ubiquitous glycosaminoglycan of the extracellular matrix and an integral part of the endothelial glycocalyx. It was found that they could be successfully used to deliver hydrophobic, poorly bioavailable compounds to the vessel wall [128].

### 4.3. ^13^C MRI

**Figure 5:**
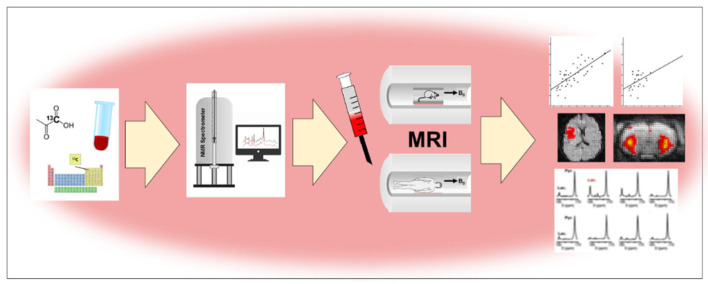
The scope of use of ^13^C MRI in research.

The implementation of ^13^C MRSI into scientific research enables the monitoring of metabolism in vivo and in real time, thus minimizing the negative impact of the test on the body, as it is a non-invasive method. The scope of use of ^13^C MRI in research is shown in Figure 5. In the case of the ^13^C study, in their studies, Johansson et al. presented the evaluation of cerebral perfusion in a rat model by ^13^C MRI after intravenous administration of 1,1-bis (hydroxymethyl) -113C-cyclopropane-D8 [129]. On the other hand, the group of Magnusson et al. used the MRI method for imaging a catheter filled with ^13^C hyperpolarized contrast agent as a new passive nonproton technique [130]. Day et al. presented a study using ^13^C MRS to test the therapeutic response in mice with lymphoma, which could be a new approach to assess the tumor response to applied therapies in clinical treatment [45]. Albers et al. presented research on non-invasive biomarkers for the detection and evaluation of prostate cancer in a mouse transgenic adenocarcinoma (TRAMP) model using a new technique based on ^13^C-labeled hyperpolarized pyruvate [131]. Dafni et al. presented the results of ^13^C MRS measurements using hyperpolarized ^13^C-pyruvate for molecular imaging [132]. In the research, the group of Lupo et al. performed 3D lactate image sequences to evaluate metabolic processes in a mouse prostate cancer model after injection of pre-polarized ^13^C-pyruvate [133]. Marjańska et al. presented in vivo metabolism studies in rat brain of ^13^C MRS hyperpolarized ^13^C-pyruvate and 2-^13^C pyruvate increasing the low sensitivity of dynamic nuclear polarization [134].

Lupo et al. in their work, presented the use of ^13^C MRS to assess metabolism in a mouse model of prostate cancer, based on the determination of changes in metabolism, the advanced stage of disease development was assessed [133]. Bhattacharya et al. performed in vivo studies on the imaging of atherosclerosis in mice with the hyperpolarizable molecule, 2,2,3,3-tetrafluoropropyl 1- (13) C-propionate-d (2,3,3) (TFPP) in the imaging of hyperpolarized ^13^C MRS, binding of TFPP to lipids resulted in the generation of a characteristic peak in MRS [135]. Hu et al. presented an in vivo study with hyperpolarized ^13^C-pyruvate MRSI to visualize glycolysis in tumor formation and regression in a Myc driven liver cancer model [136]. Whereas Hu et al. used dynamic nuclear polarization to measure the metabolism of ^13^C intracellular pyruvate and lactate in in vivo studies in rats after injection of hyperpolarized 1-^13^C alanine [137]. Bohndiek et al. used hyperpolarized [1-^13^C]-ascorbic acid (AA) and [1-^13^C]-dehydroascorbic acid (DHA) to evaluate them as redox status probes in in vivo studies [138]. The results confirmed that ^13^C hyperpolarized vitamin C can be used as a non-invasive probe in in vivo studies. Chaumeil et al. instead, presented the use of hyperpolarized ^13^C MRS imaging to monitor the progress of everolimus treatment by measuring the HP-lactate to pyruvate ratio for a glioblastoma (GBM) model in in vivo studies [139]. Keshari et al. presented an MRS study after injection of HP ^13^C as a biomarker of prostate cancer in living human tissues for the evaluation of metabolic changes in neoplastic tissue [140]. Chen et al. presented in their work studies on the determination of the therapeutic response after radiotherapy on the basis of changes in hyperpolarized lactate signals [1-^13^C]. A decrease in the signal in relation to pyruvate [1-¹³C] was observed in MDA-MB-231 tumors as early as 96 h after irradiation with ionizing radiation, which may be promising progress in the accurate monitoring of therapy progress and planning for further treatment stages [141]. Schroeder et al. presented the use of hyperpolarized ^13^C MRI to evaluate energy metabolism in the pathogenesis of heart failure by detecting early and late changes in pyruvate metabolism in in vivo studies [142]. In his work, Zhang assessed the potential of using hyperpolarized ^13^C MRI to assess cancer by determining metabolite changes, such as flux changes in multiple signaling pathways in cancer [143]. Whereas Durst et al. using dynamic nuclear polarization, tracked pyruvate bolus in rats by excitation of low-angle RF allowing collection of selective information [144]. Dzien et al. in their work, presented the use of ^13^C MRI hyperpolarization in in vivo studies to detect the expression of the transgenic activity of pyruvate decarboxylase [145]. In his work, Gordon et al. presented a method of simultaneous ^1^H and ^13^C imaging, where it was shown that simultaneous multinuclear imaging allows morphological images with ^1^H and metabolic images with ^13^C to be obtained. Moreover, the images are spatially correlated, taken at the same time [146]. On the other hand, the group of Tang et al. used a pulse sequence of 2D RF pulses in hyperpolarized ^13^C imaging to monitor bolus in in vivo broadcasts in rats. The obtained time resolution was eight times higher compared to 1D imaging [147]. Using dissolution–dynamic nuclear polarization (dissolution–DNP) ^13^C MRS, Flori et al. determined real-time cardiac metabolism in pigs in in vivo studies, the dissolution–DNP procedure of Na [1-^13^C] acetate was used as a bolus for imaging with the MR 3 T scanner [148]. In the same year, Fuchs et al., using imaging of the ^13^C chemical shift, followed the dynamics of the metabolite in plants [149]. Also, Dzien et al. presented the use of ^13^C MRS measurements with hyperpolarized [1-^13^C]pyruvate to detect the expression of transgenic pyruvate decarboxylase activity in vivo [145]. Sriram et al. investigated the ^13^C hyperpolarization of pyruvate metabolism in human renal tissue sections using an MR adapted bioreactor platform, it was shown that malignant renal cancers (RCC) have increased lactate production compared to benign lesions [150]. Park et al. presented the measurement of ^13^C-bicarbonate in a glioblastoma model and healthy brains using ^13^C MRS. The ratio of hyperpolarized ^13^C-lactate to ^13^C-bicarbonate was determined, which confirms the possibility of using this factor as an early biomarker to assess the therapeutic response [151]. Serrao et al. presented the use of ^13^C MRSI with hyperpolarized pyruvate[1-^13^C] for metabolic imaging in pancreatic cancer (PCa) in a mouse model to determine alanine and lactate concentrations and to determine the activity of lactate dehydrogenase (LDH) and alanine aminotransferase (ALT), to determine disease progression [152]. ^13^C MRS detects the ^13^C carbon isotope in the metabolites of the brain, but due to its insufficient amount in the body, to perform the test additional ^13^C is administered to the patient, and new hyperpolarization techniques allow for signal enhancement in in vivo tests [153]. Due to the implementation of the hyperpolarized HP MR technique, ^13^C molecular imaging is possible [154]. Faarkrog et al. assessed the metabolism of [1-^13^C] hepatic pyruvate in mice using the MRS method, which may contribute to the identification of impairment of specific metabolism in the liver [155]. Tang et al. used dynamic ^13^C MRI imaging for real-time bolus tracking in in vivo studies in their research [156].

### 4.4. ^31^P MRI

**Figure 6:**
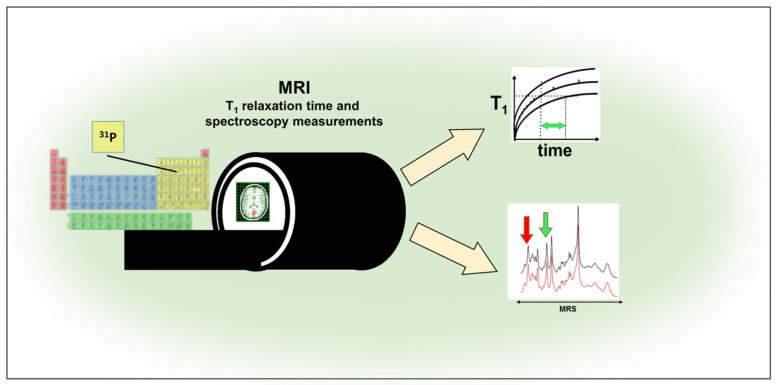
Application Range of ^31^P MRI.

The application range of the ^31^P MRI includes the determination of the T_1_ relaxation time and spectroscopic examinations (Figure 6). The use of ^31^P MRS, unlike classical proton spectroscopy, enables the detection of high-energy phosphates such as: ATP and phosphocreatine (PCr). Bhujwalla et al. using markers of dimethyl methylphosphonate (DMMP), 3-aminopropylphosphonate, P assessed the quantitative concentration of intracellular and extracellular metabolites with MRS ^31^P ap-assisted in solid tumors in vivo [157,158]. The group of de Roos et al. used the classical MR method and the ^31^P spectroscopic method, which can be combined to obtain complete cardiovascular diagnostics [158]. In their work, the group of Kemp et al. presented the evaluation of the concentration of metabolites [Pi] and [phosphocreatine (PCr)] by ^31^P MRS in muscles in in vivo studies, eliminating the problem of measuring PCr and Pi signal intensity by phantom calibration [159]. In their research, Kozerke et al. presented prospective tracking used in the ^31^P MRS of the heart [160]. On the other hand, Schneider-Gold et al. presented quantitative MRS and MRI ^31^P studies in patients with type 2 myotonic dystrophy without diagnosed heart disease, in order to determine the risk of myocardial and skeletal muscle involvement [161]. On the other hand, Lee et al. in in vivo studies in rats used superparamagnetic iron oxide nanoparticles that enable the tracking of transplanted olfactory OEC cells and the determination of their temporal and spatial migration in normal and damaged spinal cords [162]. Landis et al. presented studies of tracing transplanted hepatocytes in irradiated livers with ^31^P MRS. Transplantation of hepatocytes expressing creatine kinase (CK) allows monitoring of hepatic tissue reconstruction in transplanted cells [163]. By contrast, Wijnen et al. using the phenomenon of polarization transfer (PT) ^31^P MRI in a 9.4 Tesla apparatus, improved the specificity of the detection of phospholipids, phosphoethanolamine, and glycerophosphoethanolamine in breast cancer models [164]. On the other hand, Li et al. using ^31^P MRS, assessed the phosphate metabolite in muscles and its oxidative capacity in skeletal muscle work in women and men. The results showed differences in PCr / Pi, which was higher in men, suggesting higher energy transfer efficiency in men [165]. Layec et al. used ^31^P MRS to assess the capacity of peak mitochondrial adenosine triphosphate (ATP) in human skeletal muscle [166]. Liu et al. described the use of ^31^P MRS to evaluate tissue metabolism in in vivo studies [167]. Chouinard et al. performed ^31^P MRS studies in healthy siblings compared to patients with FEP (including schizophrenia spectrum and affective psychosis) to check bioenergy and redox status, which may be helpful in identifying and assessing the risk of psychosis [168]. Ren et al. performed in vivo studies in the 7T field of CSF and peripheral blood with the use of ^31^P MRS, the studies confirmed that in the obtained signal we are dealing with signals from other components of the human brain that should be suppressed in order to be able to assess metabolism [169]. Philips et al. in their in vivo studies, developed an endorectal coil for prostate imaging and evaluation of prostate metabolites by ^31^P MRS in the 7T field [170].

### 4.5. ^17^O MRI

**Figure 7:**
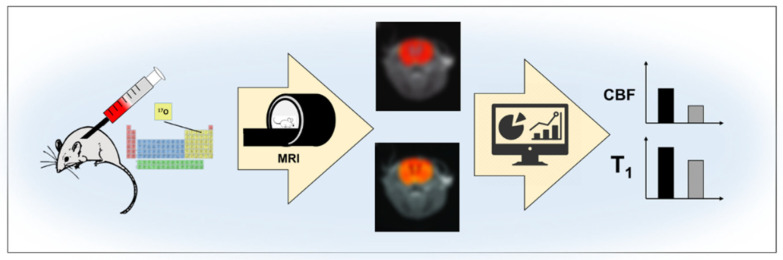
Schematic of blood-brain barrier (BBB) assessment with MRI.

^17^O MRI is used to evaluate bolus of ^17^O tracking in in vivo animal studies (Figure 7). In research on ^17^O, Zhang et al. using ^17^O MRI assessed the tracking of an intravenous bolus of ^17^O in mice studies. The blood-brain barrier (BBB) was assessed, and cerebral blood flow (CBF) was determined by MRI. The group found an increase in cerebral vascularization in the mice and decreased water exchange by the BBB. Aquaporin-4 (AQP4) has been found to play an important role in regulating water exchange [171].

## 5. ^23^Na MRI

Sodium ^23^Na MRI allows for direct imaging of sodium ions in tissue. ^23^Na is regulating osmotic pressure and ionic homeostasis at the cellular level. It has been shown that total sodium concentration is significantly higher in malignant breast lesions compared to benign lesions and healthy fibroglandular tissue [172]. ^23^Na MRI offers a critical measurement of sodium in human heart. The tissue sodium concentration in heart is about ~40 µmol/g wet weight and ^23^Na MRI is used to detect defects in sodium concentration by changes in sodium transport [173]. ^23^Na MRI has increasing potential as a biomarker for neurodegeneration and neuroinflammation in multiple sclerosis [174]

## 6. The Future Opportunities of Multinuclear MRI

In order to enhance the role of multinuclear MRI in the field of drug discovery more hardware and software advances are required to image smaller samples and lower concentrations of nuclei. Detection of nuclei other than protons can significantly expand opportunities of the MRI method. Instruments capable of ultra-high field strengths, ≥3 Tesla, are commonly engineered and have resulted in higher SNR and higher resolution images. Multinuclear MRI can provide anatomical, analytical and functional information by probing ions involved in metabolic processes at the cellular level. An increased SNR and high spatial resolution conferred by ultrahigh field MRI can be used to improve the delineation of small anatomical structures and subtle pathology.

## 7. Conclusions

Recently, there has been an increase in research in the field of cell tracking with MRI. The used combinations of drugs with contrast agents are aimed at developing a new generation of contrast agents, which would enable functional imaging of cells. MRI-based cell tracking has unique potential. However, there is a need to optimize the parameters of the therapy in order to enable clinical translation. It is anticipated that cell and drug tracing in clinical trials will be routinely used in the near future. The availability of MRI scanners will allow for the implementation of research. Today, the tracking of MRI drugs remains a challenge. In recent years, the use of drugs in MR imaging has been extensively evaluated in terms of therapeutic effects and drug delivery within the lesion. It should be noted, however, that MR imaging is a non-invasive examination and enables the acquisition of high-resolution images. The potential of multinuclear MRI is enormous in basic research and in clinical trials. Tuning MRI scanners to the Larmor frequency of heavy nuclei increases their biomedical value, and further research in this area is welcomed. In addition, multi-FID techniques improve time efficiency in data collection. They provide more information in a single survey and provide better sensitivity compared to conventional measurements.

## Figures and Tables

**Table 1:** Nuclei currently used in human MRI and MRS studies.

Nucleus	Spin (I)	(γ) MHz/T	Abundance %	Comments
^1^H	$\frac{1}{2}$	42.58	99.99	^1^H occurs in nearly all biological molecules Primary nucleus of interest for MRI and MRS
^3^He	$\frac{1}{2}$	32.43	0.0001	Hyperpolarized ^3^He is used as a gaseous contrast agent for pulmonary MRI
^13^C	$\frac{1}{2}$	10.71	1.108	Well resolved peak but week signal. Labeled substrates uses to measure metabolism
^19^F	$\frac{1}{2}$	40.06	100	Strong signal, but does not naturally occur in biological tissues, used to label drugs
^23^Na	$\frac{3}{2}$	11.26	100	Strong signal, no natural chemical shifts so only MRI (no MRS)
^31^P	$\frac{1}{2}$	17.24	100	Strong signal, important in monitoring of energy in metabolism
^129^Xe	$\frac{1}{2}$	11.78	26.44	Hyperpolarized ^129^Xe serves as a gaseous contrast agent for MRI

**γ**—gyromagnetic ratio.

**Table 2:** Hyperpolarized ^13^C MRI/MRS the main values for pulse sequence setup.

No.	Magnetic Field	Protocol	Reference
1	1.5 T	55 degree flip angle, TR=1.3 s, TE=29.7 ms	[42]
2	3 T	10 degree flip angle, TR=80 ms, TE =30 ms	[43]
3	7 T	5 degree flip angle, TR=1s, TE =30 ms,	[44]
4	9.4 T	6 degree flip angle, TR=1.5s, TE =30 ms	[45]
5	14.1 T	66 degree flip angle, TR=83, TE =30 ms	[46]

## Data Availability

Data are contained within the article.
